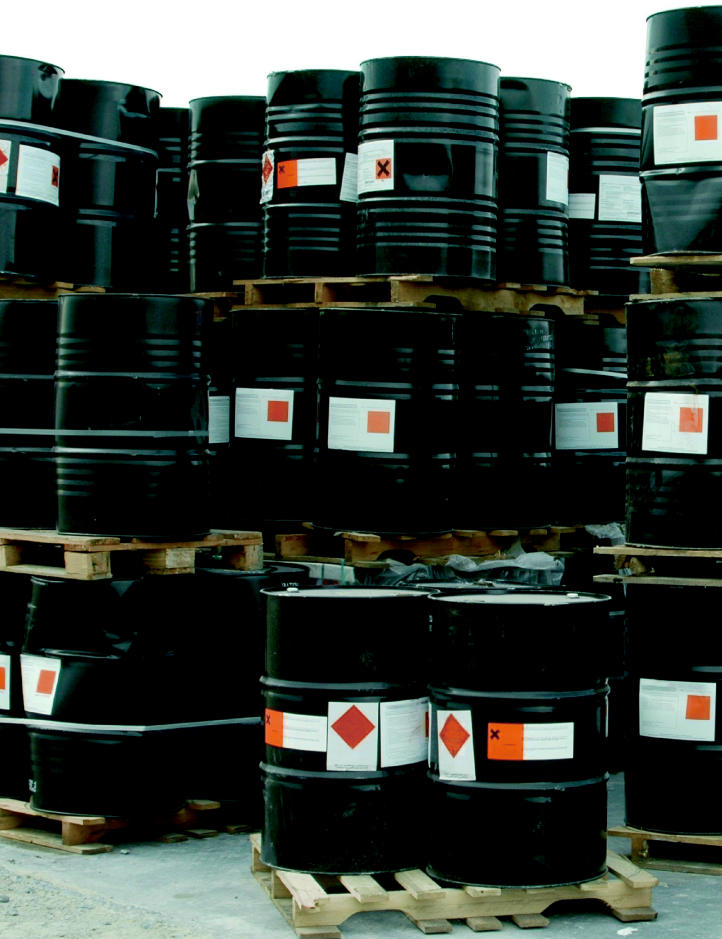# GAO Sounds Off on Chemical Regulation

**DOI:** 10.1289/ehp.113-a828

**Published:** 2005-12

**Authors:** Harvey Black

Since 1976 the Toxic Substances Control Act (TSCA) has given the federal government the power to require that chemicals are properly tested and regulated before they reach the market, and that they don’t pose unreasonable risks to human and environmental health. TSCA is the key piece of legislation governing the way the U.S. Environmental Protection Agency (EPA) reviews and regulates chemicals including solvents and constituents of paints, fuels, and plastics. Yet, concerns persist about chemical safety and the adequacy of regulation.

Now, in a June 2005 report titled *Chemical Regulation: Options Exist to Improve EPA’s Ability to Assess Health Risks and Manage Its Chemical Review Program,* the Government Accountability Office (GAO) has reviewed the EPA’s efforts to control the risks of new chemicals not yet in commerce, to assess the risks of existing chemicals used in commerce, and to publicly disclose information provided by chemical companies under TSCA. The report points out shortcomings in TSCA and its implementation, and suggests ways to strengthen the law.

## HPV Chemicals

TSCA authorized the EPA to both assess new chemicals before they enter the marketplace and to review chemicals already on the market. But when the law was enacted, thousands of chemicals already being used were grandfathered in. “Those sixty-two thousand or so chemicals were just accepted as being okay to be in commerce without any kind of EPA risk analysis,” says David Bennett, the report’s lead analyst.

Even aside from these grandfathered chemicals, the EPA has an enormous number of chemicals to examine, so the agency has narrowed its approach. “We have decided to focus our work on the high-volume chemicals, using volume as a surrogate for [human and environmental] exposure,” says Charles Auer, director of the EPA Office of Pollution Prevention and Toxics. The High Production Volume (HPV) Challenge Program was started in 1998 with the goal of looking at some 2,800 chemicals that were produced in quantities exceeding 1 million pounds per year as of 1990. The voluntary program was established by the EPA, Environmental Defense, the American Chemistry Council (ACC), and the American Petroleum Institute to identify and fill gaps in basic hazard data for these chemicals, and to make those data publicly available by 2005.

The information garnered from the HPV Challenge Program “will allow us to prioritize among the chemicals and then obtain additional information where appropriate or take control actions,” says Auer. Michael P. Walls, managing director of health, products, and science policy at the ACC, adds, “Through the [HPV Challenge] Program we provide a mechanism to assure the agency that there is adequate information on which to base current risk management decisions.”

“The program is a light-some-candles-rather-than-sit-and-curse-the-darkness initiative,” says Karen Florini, a senior attorney with Environmental Defense. “It gathers preliminary basic screening-level information. It’s clearly valuable; it’s just limited.”

Despite progress made to date, the GAO report states there are 300 chemicals in the HPV Challenge Program “for which chemical companies have not agreed to provide the minimal test data that EPA believes are needed to initially assess their risk.” Auer regards that situation as “unfinished business.” He says the EPA is developing rules to require industry to test those chemicals. “We hope to finalize that test rule at the end of this year or early next year,” he says.

Both Auer and Walls note there could be a variety of reasons for the chemical industry not doing this testing voluntarily. For example, in some instances domestic manufacturers of a chemical have said they would be willing to provide information only if foreign competitors who export the chemical to the United States would share the cost of testing—support that was not forthcoming. However, once a test rule is in effect, anyone who produces or imports the chemical must comply and provide the data.

## New Chemicals

The GAO report also voices concern about the EPA’s efforts to regulate new chemicals. Though the report noted that the EPA has taken actions to regulate exposure to about 3,500 of 32,000 new chemicals submitted for review since TSCA was enacted, the GAO has qualms about the way in which those chemicals were examined.

The EPA typically does not have enough data on a submitted chemical’s properties to determine its toxicity. Consequently, it may compare a new chemical with closely related model chemicals to predict whether the new compound will pose a safety hazard. “We found evidence that in some cases the models were not entirely predictive. The problem is that in some cases there is just not a lot of data out there to show how predictive the models are,” says Bennett.

Auer counters that the models do what they are supposed to—identify potentially hazardous candidates for further testing. He adds that the models also tend to err on the side of caution—that is, they tend to identify chemicals that appear to be hazardous but prove to be safe upon further examination.

The GAO report also notes that TSCA does not require chemical companies to submit data to the EPA on the toxicity, routes of exposure, or potential extent of exposure of new chemicals. “I think it is scandalous that new chemicals can be brought to market without being accompanied by any actual data,” Florini says. “Eighty-five percent of PMNs [premanufacture notices, which must be submitted to the EPA at least 90 days before production of a new chemical begins] are submitted with no health data, and reliable models aren’t available for many end points, particularly for long-term health effects other than cancer.”

But Walls says chemical manufacturers must be prepared to supply data to the EPA if a chemical has characteristics of persistence, bioaccumulation, and toxicity. “The assumption that new chemical applications are filed in the United States with no information is not right,” he says. He agrees that TSCA does not require safety and exposure data, but adds that if a company is submitting a chemical to which people could be exposed, the company would be “remiss” in not providing that information. Still, the data need be provided only if the agency asks for them.

Auer asserts that requiring toxicity testing before a chemical is actually manufactured, as required by TSCA, could interfere with innovation in the industry. He estimates the cost of providing the information wanted by the EPA to be in the range of a quarter of a million dollars for new chemicals. Auer says the expense of testing before a chemical company knows whether there will be a demand for a chemical could hobble efforts to develop improved chemicals, and maintains that the EPA’s track record of taking action to reduce the risk of new chemicals is a good one.

## TSCA, Take Two

Faced with the lack of required data for new chemicals and what the GAO regards as the uncertain effectiveness of the voluntary HPV Challenge Program, the report recommends that Congress give the EPA the authority to require chemical manufacturers to generate and provide test data on HPV chemicals. That recommendation is embodied in legislation introduced in Congress this summer by senators Frank Lautenberg (D–NJ) and James Jeffords (I–VT). The bill also would give the EPA the authority to require those data for all chemicals used, and to prioritize which chemicals the industry would have to test. Auer says the EPA has not yet taken a position on the bill. Environmental Defense supports it, while the ACC opposes it, saying it duplicates the EPA’s existing authority under TSCA.

The GAO offers a number of other recommendations to strengthen the act. Among them are validating and improving the models used by the EPA to assess risks of chemicals; requiring chemical companies to submit testing results of chemicals with PMNs; letting the EPA regulate chemicals if they pose a “significant” risk to health or the environment rather than the more stringent “unreasonable” risk; and setting national goals for reducing the overall use of toxic chemicals.

Auer says the EPA will soon have much better exposure information under amendments to the TSCA Inventory Update Rule, which requires chemical manufacturers to submit basic production data every four years for chemical substances (including imports) manufactured for commercial purposes in amounts of 25,000 pounds or more at a single site. According to the EPA, the 2003 amendments tailor reporting requirements to more closely match the EPA’s information needs, provide a vehicle for the EPA to obtain updated information on the potential human and environmental exposures of chemical substances listed on the TSCA Inventory, and improve the utility of the information reported under the rule.

With the amendments, “we will know the chemicals that are in consumer products,” Auer says. “We will know the number of workers that are exposed to chemicals. We will have a better basic idea of the uses of chemicals. So EPA in sixteen months will have basic exposure information on [HPV] chemicals.”

Moreover, Auer says the EPA will require the information to be updated every five years so the agency can understand how chemical use is shifting and whether safer alternatives have been developed in the meantime. He says the information will allow the EPA to identify HPV chemicals that are candidates for more testing or for enhanced regulation. Further, Walls says the chemical industry on its own initiative will supply the EPA with hazard data on around 500 chemicals that reached the high-volume threshold between 1990 and 2002.

Nevertheless, Bennett reserves judgment on the HPV Challenge Program’s effectiveness. “The process is ongoing, so I would hesitate to say how successful it is, because EPA has not received all the data that industry has promised to deliver. We won’t know for some time whether the program will be successful,” he says.

Florini echoes that view, and also points out that the EPA is “way behind” in making public the information submitted to date. “Six years into the HPV Challenge Program, it’s really regrettable that the database hasn’t yet been released, though it apparently will be out by year-end,” she says.

After three decades of existence, it is appropriate that TSCA is undergoing significant examination, as the EPA, the chemical industry, environmentalists, and legislators all look at ways to revise this major statute. What a revised TSCA will look like after this examination, however, is far from certain.

## Figures and Tables

**Figure:**